# Paradoxical and Retrograde Air Embolism from Pressurized Peripheral Bolus

**DOI:** 10.1155/2021/1063264

**Published:** 2021-10-05

**Authors:** Joshua Santucci, Naresh Mullaguri, Anusha Battineni, Raviteja R. Guddeti, Christopher R. Newey

**Affiliations:** ^1^Cerebrovascular Center, Neurological Institute, Cleveland Clinic Foundation, Cleveland, OH, USA; ^2^Division of Neurology, Department of Medicine, Prisma Health Upstate, University of South Carolina Greenville School of Medicine, Greenville, SC, USA; ^3^Division of Cardiology, Cape Fear Valley Medical Center, Fayetteville, NC, USA; ^4^Epilepsy Center, Neurological Institute, Cleveland Clinic Foundation, Cleveland, OH, USA

## Abstract

**Introduction:**

Cerebral air embolism is a rare, yet serious neurological occurrence with unclear incidence and prevalence. Here, we present a case of fatal cerebral arterial and venous cerebral gas embolism in a patient with infective endocarditis and known large right-to-left shunt and severe tricuspid regurgitation following pressurized fluid bolus administration. *Case Presentation*. A 32-year-old female was admitted to the medical intensive care unit from a long-term acute care facility with acute on chronic respiratory failure. Her medical history was significant for intravenous heroin and cocaine abuse, methicillin-sensitive *Staphylococcus aureus* tricuspid valve infective endocarditis on vancomycin, patent foramen ovale, septic pulmonary embolism with cavitation, tracheostomy with chronic ventilator dependence, multifocal cerebral infarction, hepatitis C, nephrolithiasis, anxiety, and depression. After intravenous fluid administration, she became unresponsive with roving gaze, sluggish pupils, and hypotensive requiring vasopressors. CT of the brain showed diffuse arterial and venous cerebral air embolism secondary to accidental air administration from fluid bolus. Magnetic resonance imaging of the brain showed diffuse global anoxic injury and flattening of the globe at the optic nerve insertion. Given poor prognosis, her family chose comfort measures and she died.

**Conclusions:**

Fatal cerebral air embolism can occur through peripheral intravenous routes when the lines are inadequately primed and fluids administered with pressure. Caution must be exercised in patients with right-to-left shunting as air may gain access to systemic circulation.

## 1. Introduction

Cerebral air embolism is a rare, yet serious neurological occurrence with unclear incidence and prevalence. Reportedly, somewhere around 20,000 cases of cerebral air embolism occur each year [1], often presenting as an arterial or venous embolism, but usually not both. Many cerebral air embolism cases present from an iatrogenic cause, such as central venous access insertion or removal, surgical procedures particularly neurosurgical and cardiothoracic procedures, or trauma [2–9]. Additionally, arterial emboli can be seen from venous air introduction, especially if an intracardiac shunt is present, as a paradoxical embolus [2, 3, 10–18]. Here, we present a case of fatal cerebral arterial and venous cerebral gas embolism in a patient with infective endocarditis and known large right-to-left shunt and severe tricuspid regurgitation following pressurized fluid bolus administration.

## 2. Methods

This article is a case report with the pertinent literature review. Various terms were searched on the PubMed database for the literature review including air embolism, gas embolism, patent foramen ovale (PFO), tricuspid regurgitation, anoxic injury, acute ischemic stroke, and paradoxical embolism.

## 3. Case Presentation

**Figure 1:**
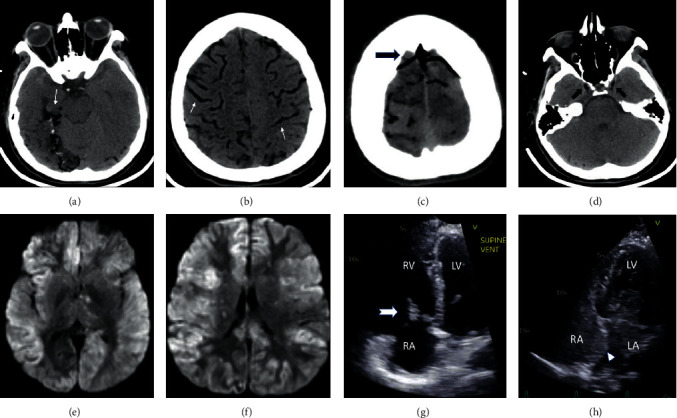
(a, b) Computerized tomography (CT) of the brain axial sections showing air embolism in the cortical branches of the posterior cerebral, middle, and anterior cerebral arteries, respectively (white arrows). (c) Same CT scan showing air in the superior sagittal sinus and cortical veins (black arrow). (d) CT scan showing air in the bilateral cavernous sinus (black arrow). (e, f) Diffusion-weighted sequence of a magnetic resonance image of the brain showing diffuse bilateral cortical anoxic injury. (g, h) TTE showing tricuspid valve vegetation (white arrow) and location of interatrial septum and PFO, respectively (white arrowhead). Bubbles in the left atrium and ventricle that crossed within 3 cardiac cycles.

A 32-year-old female was admitted to the medical intensive care unit from a long-term acute care facility with a chief complaint of acute on chronic respiratory failure. Her medical history is significant for intravenous heroin and cocaine abuse, methicillin-sensitive *Staphylococcus aureus* tricuspid valve (TV) infective endocarditis on vancomycin, PFO, septic pulmonary embolism (PE) with cavitation, tracheostomy with chronic ventilator dependence, multifocal cerebral infarction, hepatitis C, nephrolithiasis, anxiety, and depression. She was requiring 100% oxygen with high positive end-expiratory pressure (PEEP) (20 mm Hg) on admission. Her Glasgow Coma Scale was 15 on admission. Sputum cultures and blood cultures were obtained, and she was started on broad-spectrum antibiotics. Computerized tomography (CT) of the chest was unremarkable for PE, left lower lobe collapse, and improving lung cavitation. She was being evaluated for valve and PFO repair. Transthoracic echocardiogram showed normal ejection fraction, dilated right ventricle, elevated right ventricular systolic (45 mm Hg), and right atrial pressures (15 mm Hg), along with 4+ tricuspid regurgitation with a highly mobile TV vegetation (Figure 1(g)). There was a large right-to-left shunt through the PFO on bubble study with spontaneous opacification of the left atrium and ventricle (Figure 1(h)). Her ventilator requirements improved on decreased PEEP at 10 mm Hg, and her oxygen requirement improved to 60% as her right-to-left shunting improved.

On day three, her oxygen requirements increased and required inhaled epoprostenol with a good response. On day four, she developed hypotension with blood pressure of 78/43 mm Hg. She was resuscitated with one liter of intravenous lactated ringers with a pressure bag into a midline peripheral access at 23:15 pm. Bolus was finished at 12:00 am, and the patient's neurological exam was unremarkable. At 00:25 am, she became unresponsive with a roving gaze, sluggishly reactive pupils, decerebrate posturing, cyanotic with persistent hypotension requiring vasopressors. After stabilizing, a CT scan of the brain was obtained which showed diffuse arterial and venous cerebral air embolism (Figures 1(a)–1(d)). Air embolism was thought to be secondary to accidental administration of air while administering fluid bolus. She was laid flat, and 100% oxygen was administered. Air embolism resolved by the next day on the follow-up CT scan of the brain, but her neurological exam did not improve. On day six, her pupils were fixed and nonreactive. She was managed medically with hyperventilation, hyperosmolar therapy, and with head elevation. Magnetic resonance imaging (MRI) of the brain showed global anoxic injury and flattening of the globe at optic nerve insertion suggestive of intracranial hypertension (Figures 1(e)-1(f)). She subsequently developed diabetes insipidus, which was managed with vasopressin infusion. Given her poor prognosis, the family opted for comfort measures, and she died on the eighth day.

## 4. Discussion

**Figure 2:**
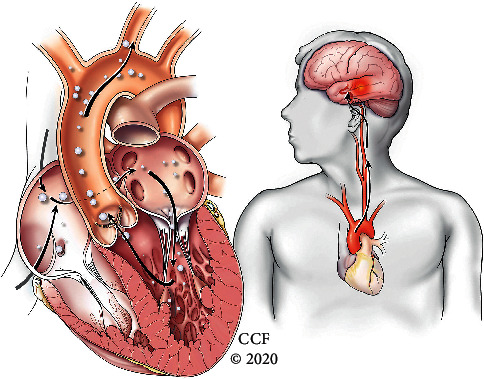
Arterial air emboli: pathway of air emboli paradoxically travelling from the right atrium across an intracardiac shunt into the left atrium and subsequently into the left ventricle and aorta before entering the carotid and then intracranial arteries, thereby causing air emboli to enter the brain.

**Figure 3:**
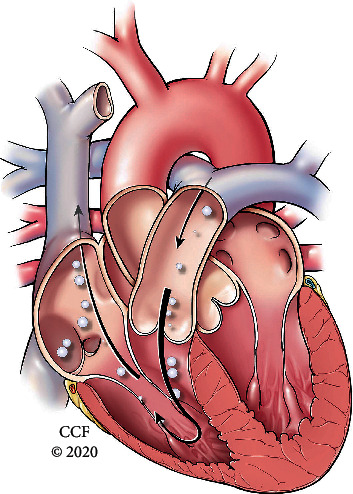
Venous air emboli: proposed mechanism where air travels in a retrograde fashion secondary to severe tricuspid regurgitation forcing air back into the superior vena cava where it ascends into the jugular veins and intracranial venous structures.

Iatrogenic air embolism can be a rare and serious cause of brain injury. In patients with a right-to-left shunt (PFO, septal defect, and pulmonary AV malformation), pressurized fluid administration through peripheral intravenous lines may cause paradoxical air embolism. In our patient, the pathway of paradoxical air embolism was likely via a large PFO. However, confounding this is the history of bilateral pulmonary cavitary lesions with hydropneumothorax being a potential nidus for a pulmonary AV shunt; however, PFO is favored more likely given early shunting within the first few heartbeats during the echocardiogram. Our patient had both arterial (ACA, MCA, and PCA) and venous (superior sagittal sinus and cavernous sinus) air emboli noted on imaging. The mechanism for the arterial embolism is paradoxical via venous air entry through the peripheral IV line crossing the PFO to gain entry into the arterial circulation (Figure 2). The proposed mechanism for the venous air emboli is less certain, but was thought to be secondary to severe tricuspid regurgitation driving retrograde pressure into the superior vena cava and subsequent jugular veins (Figure 3). The lack of valves in the jugular veins allows the air to enter the venous circulation more easily [19]. This phenomenon is thought to be underestimated in the reported literature. Additional reported pertinent factors include smaller bubble size, lower specific gravity of air compared to blood, vein diameter, cardiac output, and increased intrathoracic pressure [2, 19–22]. Further suggested pathophysiology is whereby venous gas emboli enter the pulmonary arterial circulation causing increased afterload and subsequent strain of the right heart leading to reduced preload of the left heart causing overall cardiovascular collapse resulting in hypotension and hypoxemia. As a result, this causes reduction in anterograde cardiac flow and impaired venous return to the right atrium, superior vena cava stagnation, and eventual venous embolism [23].

Indeed, the literature review on both venous and arterial embolization secondary to iatrogenic venous air injection in the setting of PFO is not well reported, and secondary to tricuspid regurgitation is, to these authors' knowledge, not reported. Tsetsou et al. reported 5 cases of cerebral air embolism, 4 of whom had PFO detected on echocardiogram [10]. Heckmann et al. reported 6/15 patients with PFO and 9/15 with pulmonary shunt as the source of right-to-left shunting [11]. In these studies, however, none of the patients had similar mechanisms of iatrogenic insult that our patient did. Bowles et al. reported paradoxical air embolism in a patient who underwent pulmonary artery catheter removal [12]. Various case reports exist wherein cerebral air embolism occurred secondary to central venous access removal, some with right-to-left shunting and others without [15–18, 24, 25]. Harlan et al. even reported a spontaneous air embolism in a patient with a pulmonary arteriovenous malformation [26]. There are 3 case reports in the literature of air emboli causing infarction secondary to IV fluid administration via a peripheral IV line [2, 21, 22]. One similar case was found in the literature reported by Mendenhall et al. whereby a patient who had a motor vehicle accident suffered cerebral infarction from arterial air embolism after resuscitation of IV fluids with a pressure bag via an antecubital IV line with presence of a PFO [14]. Overall, there does appear to be around a few hundred cases reported of iatrogenic cerebral air embolism, as seen in a systematic review by Hatling et al. [3]. Otherwise, many reports exist wherein iatrogenic air emboli have occurred secondary to central venous catheter removal, endoscopic procedures, trauma, deep sea diving, and surgical procedures, especially upright neurosurgical or otorhinolaryngological procedures [2–9]. The presence of a PFO in these cases is not as well described. A brief summary of the most pertinent cases in the literature with reported intracranial imaging and echocardiogram findings as well as our case is given in Table 1.

Overall, air embolism should be considered especially important, given the known prevalence of PFO in the general population and high rate of devastating consequences. Earlier studies reported 80–90% morbidity and mortality, but this number has been more recently reported at closer to 21% given earlier recognition and treatment when air embolism suspected [27]. Higher volumes of embolic air, rate of embolic air accumulation, importance of affected cerebral territory, focal motor deficits (especially hemiparesis), presence of Babinski sign, presence of gyriform air, initial disturbance of consciousness, older age, and retrograde ascension of venous air have all been associated with worse prognosis [20, 27–29]. Precautions should be taken during placement and removal of central and even peripherally placed IV lines (avoiding any air entry into the line itself, Trendelenburg position when placing IJ or subclavian lines, Valsalva during line removal).

## 5. Conclusions

Fatal cerebral air embolism can occur in patients through peripheral intravenous routes when the lines are inadequately primed and fluids administered with pressure. Caution must be exercised in patients with right-to-left shunting as the air may gain access to systemic circulation.

## Figures and Tables

**Table 1:** Characteristics of patients with iatrogenic air embolism with cardiac defects in the literature.

Patients	Age (years)	Echo findings	Cause of emboli	Imaging findings	Arterial embolism	Venous embolism	Long-term outcome
1^ [2]^	81	Small PFO	Peripheral line access and IV fluid administration	R parietooccipital air with subsequent stroke	Not reported	Not reported	L homonymous hemianopsia and L arm weakness
2^ [5]^	46	No PFO	Unknown	Intravascular air, edema, and tonsil herniation	Bilateral cerebral arteries	None	Death
3^ [10]^	77	Incompletely closed PFO	Bubble study during TTE	Chronic cerebellar stroke	Not reported	Not reported	3-month mRS: 1
4^ [10]^	49	PFO with massive shunt	Bubble study during TTE	Normal	Not reported	Not reported	3-month mRS: 0
5^ [10]^	67	PFO with moderate shunt	Dialysis through CVC with presumed air through PFO	Air bubbles in R MCA cortex with edema	Right MCA	Not reported	3-month mRS: 6
6^ [10]^	34	PAVMs with massive shunt and no PFO	Air entry directly into PAVMs	Air bubbles in L MCA cortex, bihemispheric strokes	Left MCA	Not reported	3-month mRS: 2
7^ [10]^	74	PFO with minimal shunt	Air entry in pulmonary veins during lung biopsy	Bihemispheric strokes	Not reported	Not reported	3-month mRS: 1
8^ [12]^	54	PFO	Removal of the PA catheter	Normal	Not reported	Not reported	No deficits
9^ [14]^	49	PFO	IV fluids pressure bagged into peripheral IV line	R occipital stroke	Right PCA	None	Not reported
10^ [15]^	76	PFO	Removal of CVC	R frontal sulcus air and infarct	Not reported	Not reported	No deficits
11^ [16]^	57	PFO	Right IJV line removal	Cerebral air embolism	Not reported	Not reported	Residual L hemiparesis
12^ [17]^	43	PFO	Right IJV line removal	Multiple embolic strokes	Not reported	Not reported	Residual L hemiparesis
13^ [18]^	49	PFO	Right IJV line removal	Air in subarachnoid vessels	Subarachnoid vessels	Not reported	No deficits
14^ [21]^	83	Not reported	Fluid bolus into peripheral IV	Air in superior ophthalmic vein	Not reported	Superior ophthalmic vein	Not reported
15^ [22]^	Not reported	Not reported	Injection into peripheral IV	Air in cavernous sinus	Not reported	Cavernous sinus	Not reported
16^ [25]^	89	No PFO	Right IJV line removal	Air in bilateral cavernous sinus	None	Bilateral cavernous sinus	No deficits
17^ [26]^	46	PAVM	High intrathoracic pressure breathing against obstructed airway	Air emboli in R hemisphere	Not reported	Not reported	No deficits
18^ [27]^	51	No PFO	Left subclavian vein line removal	Air in bilateral cavernous sinus	None	Bilateral cavernous sinus	No deficits
Our patient	32	PFO and tricuspid regurgitation	IV fluids pressure bagged into peripheral IV line	Air in SSS, bilateral ACA, MCA, and PCA; diffuse anoxic injury	Bilateral ACA, MCA, and PCA	SSS, bilateral cavernous sinus	Death

PFO, patent foramen ovale; IJV, internal jugular vein; mRS, modified Rankin Score; PCA, posterior cerebral artery; PA, pulmonary artery; MCA, middle cerebral artery; TTE, transthoracic echocardiogram; PAVM, pulmonary arteriovenous malformation; CVC, central venous catheter; ACA, anterior cerebral artery; R, right; L, left.

## Data Availability

No data were used to support this study.
